# Arsenic and Environmental Health: State of the Science and Future Research Opportunities

**DOI:** 10.1289/ehp.1510209

**Published:** 2015-11-20

**Authors:** Danielle J. Carlin, Marisa F. Naujokas, Karen D. Bradham, John Cowden, Michelle Heacock, Heather F. Henry, Janice S. Lee, David J. Thomas, Claudia Thompson, Erik J. Tokar, Michael P. Waalkes, Linda S. Birnbaum, William A. Suk

**Affiliations:** 1 Superfund Research Program, National Institute of Environmental Health Sciences (NIEHS), National Institutes of Health (NIH), Department of Health and Human Services (DHHS), Research Triangle Park, North Carolina, USA; 2 MDB, Inc., Durham, North Carolina, USA; 3 Human Exposure & Atmospheric Science Division, National Exposure Research Laboratory, U.S. Environmental Protection Agency (EPA), Research Triangle Park, North Carolina, USA; 4 National Center for Computational Toxicology, and; 5 National Center for Environmental Assessment, Office of Research and Development (ORD), U.S. EPA, Research Triangle Park, North Carolina, USA; 6 Integrated Systems Toxicology Division, National Human and Environmental Health Effects Research Laboratory, ORD, U.S. EPA, Research Triangle Park, North Carolina, USA; 7 Population Health Branch, and; 8 National Toxicology Program, NIEHS, NIH, DHHS, Research Triangle Park, North Carolina, USA; 9 NIEHS, NIH, DHHS, Research Triangle Park, North Carolina, USA

## Abstract

**Background:**

Exposure to inorganic and organic arsenic compounds is a major public health problem that affects hundreds of millions of people worldwide. Exposure to arsenic is associated with cancer and noncancer effects in nearly every organ in the body, and evidence is mounting for health effects at lower levels of arsenic exposure than previously thought. Building from a tremendous knowledge base with > 1,000 scientific papers published annually with “arsenic” in the title, the question becomes, what questions would best drive future research directions?

**Objectives:**

The objective is to discuss emerging issues in arsenic research and identify data gaps across disciplines.

**Methods:**

The National Institutes of Health’s National Institute of Environmental Health Sciences Superfund Research Program convened a workshop to identify emerging issues and research needs to address the multi-faceted challenges related to arsenic and environmental health. This review summarizes information captured during the workshop.

**Discussion:**

More information about aggregate exposure to arsenic is needed, including the amount and forms of arsenic found in foods. New strategies for mitigating arsenic exposures and related health effects range from engineered filtering systems to phytogenetics and nutritional interventions. Furthermore, integration of omics data with mechanistic and epidemiological data is a key step toward the goal of linking biomarkers of exposure and susceptibility to disease mechanisms and outcomes.

**Conclusions:**

Promising research strategies and technologies for arsenic exposure and adverse health effect mitigation are being pursued, and future research is moving toward deeper collaborations and integration of information across disciplines to address data gaps.

**Citation:**

Carlin DJ, Naujokas MF, Bradham KD, Cowden J, Heacock M, Henry HF, Lee JS, Thomas DJ, Thompson C, Tokar EJ, Waalkes MP, Birnbaum LS, Suk WA. 2016. Arsenic and environmental health: state of the science and future research opportunities. Environ Health Perspect 124:890–899; http://dx.doi.org/10.1289/ehp.1510209

## Introduction

Inorganic and organic arsenic compounds continue to pose environmental public health challenges for hundreds of millions of people worldwide (WHO 2008). Nearly every organ in the body can be affected by arsenic exposure, with health effects ranging from skin lesions and cancer to diabetes and lung disease (Naujokas et al. 2013; NRC 2014). Given the ubiquitous nature of arsenic in the environment combined with growing evidence of health effects at lower levels of exposure to arsenic than previously thought (NRC 2014), the prevention and mitigation of arsenic-induced adverse health outcomes requires more vigorous pursuit. A literature search of ongoing research related to arsenic in the environment resulted in > 1,000 papers published annually with “arsenic” in the title. From this voluminous wealth of information, the question becomes, what are the outstanding issues that would best drive future research directions?

The National Institutes of Health’s National Institute of Environmental Health Sciences (NIEHS) Superfund Research Program (SRP) (NIEHS 2015) posed this question to leading arsenic researchers in remediation, exposure, and biomedical sciences. During March–June 2014, the NIEHS hosted a workshop and webinar series, “Health Effects and Mitigation of Arsenic: Current Research Efforts and Future Directions,” in Research Triangle Park, North Carolina. This workshop and webinar series provided forums to discuss state-of-the-science and knowledge gaps in arsenic research. This review is a discussion of highlights of cutting-edge research, data gaps, and suggestions for future research directions based on discussions at the workshop (NIEHS 2014).

### Understanding Arsenic Speciation and Exposure Sources

A substantial amount of research has focused on exposure to arsenic via drinking water; however, more research is now being directed toward characterizing arsenic exposures from other sources. To develop a more complete understanding of arsenic exposure, more studies are needed to identify, quantify, and characterize arsenic in diet, soil, dust, and air. In addition, although much is known about some inorganic and organic forms of arsenic, data gaps in understanding exposures and toxicokinetics of other arsenic species (e.g., arsenoproteins, arsenolipids, and thiolated arsenic compounds) need to be addressed.

***Understanding arsenic speciation.*** Arsenic exists in many different inorganic and organic forms, and in different oxidation or valence states. The valence states of arsenic compounds relevant to human health are the trivalent (As^III^) and pentavalent (As^V^) states. These arsenic species include arsenates (compounds containing AsO_4_^3–^), arsenites (compounds containing AsO_3_^3–^), and the monomethyl (MMA) and dimethyl (DMA) metabolites. Arsenic species in the trivalent state including arsenous acid (commonly arsenite), monomethylarsonous acid (MMA^III^), and dimethylarsinous acid (DMA^III^) are generally considered more toxic at lower doses than other arsenic species (ATSDR 2007; Drobna et al. 2009), although the complexity of arsenic species interconversion and the number of uncharacterized species casts uncertainty in adherance to this generalization (ATSDR 2007).

There are numerous other arsenic species, many of which we know little about. For example, fish and algae contain arsenobetaine (C_5_H_11_AsO_2_) as well as arsenoproteins, arsenolipids, and arsenosugars (Feldmann and Krupp 2011; Schmeisser et al. 2006), many of which have not been characterized. Arsenobetaine is generally considered to be of low toxicity compared with some forms of inorganic arsenic (Leffers et al. 2013; Taylor et al. 2013). Some arsenosugars have been shown to be bioaccessible and metabolized in humans, and limited studies demonstrate toxicity *in vitro* (Feldmann and Krupp 2011; Leffers et al. 2013). Because seafood can contain up to 100 times more total arsenic than rice does, and contains a variety of poorly understood organoarsenical compounds, researchers are calling for more detailed studies of these forms of arsenic (Feldmann and Krupp 2011; Molin et al. 2015). Thiolated arsenosugars have been identified as being generated in the human gut and readily absorbed by gut epithelium (DC.Rubin et al. 2014); toxicity studies are suggestive of adverse health effects but studies are sparse (Ebert et al. 2014). In addition to toxicity differences, arsenic species can vary tremendously in terms of bioavailability, environmental fate, and transport characteristics, and remediation strategy effectiveness (Campbell and Nordstrom 2014; Gupta et al. 2012).

Future arsenic research needs to expand our understanding of the variety of arsenicals that exist in the environment, better characterize more arsenic species (e.g., currently uncharacterized arsenoproteins, arsenosugars, and arsenolipids), understand their toxicokinetics *in vivo* in humans and rodents, and evaluate their fate and transport in the environment. Furthermore, rather than measuring only total arsenic, researchers are moving toward more frequently measuring specific arsenic species in environmental and human samples to gain a more complete and detailed understanding of arsenic exposures and health risks. Researchers are exploring ways to overcome challenges in human sample collection, sample handling variability, and the short half-life of some arsenic species in solution (García-Salgado and Quijano 2014; NIEHS 2014).

***Understanding arsenic exposure from diet.*** In some regions of the world including sites in the United States, arsenic exposures from drinking water are an urgent concern in the face of high concentrations of naturally occurring arsenic (Naujokas et al. 2013). Although exposure from drinking water remains a major concern, recent research reveals other sources of arsenic exposure. Identifying and characterizing these sources of arsenic exposure is very important for finding ways to minimize exposures and health risks.

One non–drinking-water source of arsenic exposure that is of increasing concern is the diet. Arsenic is present in a wide variety of foods including fish and rice (Jackson et al. 2012; Schoof et al. 1999; Tao and Bolger 1999; WHO 2011). Fish contain high amounts of organic arsenic compounds, predominantly arsenobetaine (Molin et al. 2015; Tao and Bolger 1999). In contrast, rice contains predominantly inorganic arsenic (Jackson et al. 2012). The relative contribution of the diet as a source of arsenic exposure may be substantial, particularly when drinking-water arsenic levels are low. For example, one study (Kurzius-Spencer et al. 2014) modeled dietary exposure data that was collected in three U.S. population studies: National Human Exposure Assessment Survey (NHEXAS-AZ) (Lebowitz et al. 1995), Binational Exposure Assessment Survey (BAsES, Arizona population only) (Roberge et al. 2012), and National Health and Nutrition Examination Survey (NHANES) (CDC 2015). The authors estimated that diet contributed 54–85% of total inorganic arsenic intake for individuals whose tap water contained < 10 μg/L arsenic (Kurzius-Spencer et al. 2014). A separate analysis of data from the NHEXAS-AZ and Arizona Border Survey population studies also suggests that dietary arsenic concentrations may be better predictors of urinary arsenic than drinking-water concentrations (Kurzius-Spencer et al. 2013).

It is challenging to quantify dietary exposure by measuring arsenic in foods. A duplicate diet study is one approach that uses direct measurements of duplicate samples of the foods that study participants consume over a period of time during the study (Thomas et al. 1997). These types of studies are the most accurate because they account for individual variability in food samples due to factors such as food growing conditions, preparation methods, and modifications through processing. Although duplicate diet studies have been informative for assessing dietary arsenic exposure and estimating health risks (Saipan and Ruangwises 2009), they are expensive to conduct.

An indirect measure of arsenic in foods relies on databases that contain arsenic concentration measurements for a wide variety of food types, and then using these measurements to estimate exposure based on mean values from that data. For example, arsenic exposure from rice would be estimated based on the amount consumed and the average amount of arsenic in rice reported in these databases. One such database is the ongoing U.S. FDA (Food and Drug Administration) Total Diet Study that measures about 800 contaminants and nutrients in foods present in the average U.S. diet (FDA 2015). There are a limited number of total diet studies that have measured arsenic in foods, and many of these studies measured only total arsenic concentrations (Chung et al. 2014; Schoof et al. 1999; Tao and Bolger 1999). Furthermore, arsenic concentrations in multiple samples of the same food are highly variable, and as discussed above, making generalizations of arsenic content in a specific food can be quite difficult (Lynch et al. 2014).

One study (Kurzius-Spencer et al. 2013) compared measured urinary arsenic concentrations with modeled dietary exposure estimates based on two sets of dietary data: *a*) the results of a duplicate diet study that measured total arsenic in duplicate diet samples, water, and urine over a 24-hr period for 252 people in the NHEXAS-AZ and Arizona Border Survey studies; and *b*) a total diet study using 24-hr diaries that estimated average arsenic content in food items from several published food surveys. The researchers found that the total diet study greatly underestimated dietary arsenic intake and that the duplicate diet study more accurately reflected the amount ingested to urinary biomarkers of exposure. More research is needed to unravel the complexities of dietary arsenic exposure assessment in order to better understand this exposure pathway.

***Understanding arsenic exposure from dust, soil, and air.*** Arsenic exposure from dust, soil, and air should be better quantified and characterized, particularly near former mining sites, smelting sites, and industrial areas, including Superfund sites (Beamer et al. 2014; Menka et al. 2014; Taylor et al. 2014). A Superfund site is a location in the United States that has been contaminated with hazardous waste and identified as a priority site for cleanup by the U.S. EPA (Environmental Protection Agency) because it poses a significant risk to human health and/or the environment (U.S. EPA 2015b). The migration of arsenic from sediments and soils to groundwater sources and agricultural crops is not well understood and requires more research. For example, although a recent study in Cambodia reported that geochemical soil characteristics may be more predictive of arsenic content in rice crops than the concentrations of arsenic in water used for irrigation (Seyfferth et al. 2014), it has also been shown that high arsenic concentrations in irrigation water can increase arsenic concentrations in rice and reduce rice crop yields (Duxbury and Panaullah 2007). These types of exposure risks require better characterization, especially under special exposure scenarios such as populations who rely on rice for a large proportion of their diet and those who live near a Superfund site.

Bioavailability is another important factor to consider in allocating exposure to different sources, and ongoing research is focusing on development of cost-effective methods to measure bioavailability. For example, only a portion of the total arsenic in soil is bioavailable, or able to be absorbed, by living organisms (Juhasz et al. 2006). Arsenic bioavailability has been measured directly using expensive *in vivo* animal feeding studies called relative bioavailability assays (Rees et al. 2009). Recently, a less expensive *in vivo* assay has been developed using a mouse model (Bradham et al. 2013). Bioavailability has also been estimated using inexpensive *in vitro* bioaccessibility assays (IVBA) under conditions that mimic stomach and gastrointestinal environments, and several IVBA assays have demonstrated consistency in predicting bioavailability (Bradham et al. 2011; Brattin et al. 2013; Denys et al. 2012; Juhasz et al. 2015). One study performed extensive validity testing of 10 *in vitro* assays by comparing those results with swine *in vivo* assays using linear regression analysis, goodness of fit, variability in model bias and prediction error, and other parameters; validated studies had goodness-of-fit (*R* ^2^) values ranging from 0.59 to 0.71 (Juhasz et al. 2015). Although promising, these *in vitro* assays need to be tested further using a wider variety of sample types and larger numbers of samples. Although bioavailability testing has focused primarily on soils, more research is also warranted for bioavailability assessment of other exposure media such as dust and foods (Alava et al. 2015; Juhasz et al. 2006; Menka et al. 2014).

## Exposure Assessments and Aggregate Exposures

Assessing arsenic exposure is complex because arsenic is found in multiple forms and in multiple exposure media. The media themselves also are complex, containing other co-contaminants and microbes that can influence arsenic metabolism, bioavailability, and health effects. Aggregate exposure refers to the totality of all of these exposures and may better reflect actual exposure (Kurzius-Spencer et al. 2014). For this reason, future research aims to more thoroughly identify and characterize arsenic content as well as co-contaminants such as cadmium and fluoride in exposure media. Furthermore, understanding dynamic influences of co-exposures on the bioavailability and toxicokinetics of arsenic is very important for understanding the relationship between external dose, internal dose, and health outcomes.

### Assessment Methods for Acute and Chronic Arsenic Exposure

Concentrations of arsenic and its metabolites in biological samples, such as urine, blood, toenails, and hair, are used as biomarkers of arsenic exposure (Davis et al. 2014; Marchiset-Ferlay et al. 2012; Yu et al. 2014). Although biomarkers are very important for exposure assessment, questions remain pertaining to the relationship between biomarkers and internal exposure. For example, variability in renal function and urinary creatinine levels add uncertainties to associations between urine arsenic concentrations and internal exposure; measuring arsenic in exfoliated urinary bladder epithelial cells may reduce some of these uncertainties (Currier et al. 2014; Hernández-Zavala et al. 2008). These biomarkers are generally understood to represent different time frames of exposure (e.g., urinary arsenic for acute and recent exposures, and toenail arsenic for exposures over several months) (Marchiset-Ferlay et al. 2012). Researchers are increasingly measuring arsenic in toenails because these samples are less susceptible to variability in sample handling and easier to transport from the field to the laboratory (Davis et al. 2014; Yu et al. 2014). More recently, studies have shown associations between arsenic exposure and epigenetic modifications of specific genes, suggesting that epigenetic modifications may be useful as biomarkers of exposure (Broberg et al. 2014; Gribble et al. 2014; Koestler et al. 2013).

There are a plethora of studies linking specific biomarkers of exposure with health effects, but questions remain. New research needs to probe whether these biomarkers and exposure modeling estimates truly reflect internal exposures. For example, factors that modify arsenic metabolism *in vivo* (e.g., folate content in diet and the gut microbiome) may result in differences in metabolism and absorption, adding complexity to relationships between urinary arsenic levels and internal exposure estimates (Hall and Gamble 2012; Lu et al. 2014a, 2014b). Sample handling variability also introduces uncertainties in exposure estimates; some arsenic metabolites are more easily oxidized in urine than other metabolites (Gong et al. 2001). Urinary creatinine, conventionally thought of as a standard to normalize urine dilution between samples, may vary with arsenic-related kidney effects, age, and other factors; therefore, researchers have suggested using specific gravity to normalize for urine dilution (Peters et al. 2014; Yassine et al. 2012).

To address challenges in sample handling and environmental arsenic detection, researchers are developing systems that are more affordable and easy to use for field testing of samples (Kaur et al. 2015). One example is a portable monitor for on-site measurement of arsenic species in urine samples that is being developed by Geiner Inc. (Dwiek B, personal communication; NIEHS 2014). The system allows for rapid analysis of As^III^ and As^V^ with sensitivity down to 1–5 parts per billion. Another promising approach uses a transcriptomics platform to screen for arsenic-induced gene expression in certain bacteria and fungi as sensors of arsenic in biological samples (Rosen B, personal communication; NIEHS 2014). Once specific genes and organisms are identified, they may be useful as sensors in future rapid, portable testing systems. Exposure to arsenic also can occur indoors from dust, and a new passive sampler device provides a low-cost method for assessing indoor air exposure (Beamer et al. 2014).

More data are needed to understand relationships between exposures and biomarkers for a greater variety of exposure media and biological tissues. For example, changes in metabolomic profiles may be related to arsenic exposure and may be early indicators of adverse health effects (Martin et al. 2015; Zhang et al. 2014). There is also a substantial need to develop guideline levels for chronic exposure in different media based on toenail arsenic concentrations. Toenail samples are increasingly used for biomonitoring because they are stable and relatively easy to collect, store, and transport. It is also very important to perform speciation analysis when assessing exposure in environmental or biological samples, and tools such as the novel assay and monitoring systems described above will facilitate collection of that data. A combination of urine concentrations (measured over time), toenail concentrations, external exposure measurements, and probabilistic modeling based on intake source concentrations (e.g., diet) may be the best approach to measure aggregate exposure.

### Complex Co-exposures Associated with Arsenic

Elucidating arsenic-related health outcomes from environmental exposure is confounded by co-exposure to other agents such as lead, cadmium, fluoride, polyaromatic hydrocarbons, and pesticides (Andrade et al. 2015; Estrada-Capetillo et al. 2014; Flora et al. 2014; Huang et al. 2013). For example, groundwater with high concentrations of arsenic often naturally contains high concentrations of fluoride (Amini et al. 2008). Exposure to high levels of fluoride over long periods of time has been shown to affect bone health and other organ systems in the body (Barbier et al. 2010; NRC 2006). Some studies have shown that co-exposure of arsenic and fluoride can be synergistic or antagonistic, depending on the outcome being assessed. One study in mice demonstrated reduced oxidative stress in liver and kidney when arsenic and fluoride were administered together compared with each alone (Mittal and Flora 2007). Another study in rats found learning and memory was impaired whether exposed to both arsenic and fluoride together or separately. However, exposure to arsenic and fluoride together resulted in a more stubstantial decrease in gluatamate receptor 5 (mGluR5) mRNA expression in the cortex and mGluR5 protein expression in the hippocampus than when rats were exposed to arsenic alone; fluoride exposure alone had no significant effect on these parameters (Jiang et al. 2014). More studies are clearly needed to better understand possible effects of co-exposures. It is clear that co-exposure to fluoride and other contaminants is an important factor to consider in epidemiological studies of arsenic-related toxicity.

### Role of the Microbiome in Arsenic Metabolism and Exposure Assessment

The microbiome, particularly within the digestive tract, plays an active role in health and disease (Shreiner et al. 2015). Recent studies have been exploring relationships between the gut microbiome and arsenic exposure, metabolism, and toxicity. One recent study demonstrated that arsenic exposure of mice at environmentally relevant doses (10 mg/L in drinking water) changed the types of microbes present in the gut as well as the global metabolomic profile of those microbes (Lu et al. 2014a). In fact, about 400 microbial metabolic changes were noted in feces of the exposed mice. Also, arsenic metabolite profiles in mice changed when the gut microbiome was altered by infection or in the absence of interleukin (IL)-10 in the host (Lu et al. 2013, 2014b). Microbes from the human gut have been shown to generate thiolated arsenic metabolites, and the toxicity of these metabolites is not well characterized (DC.Rubin et al. 2014).

Together these data demonstrate potential influences of the microbiome on arsenic metabolism, as well as arsenic effects on microbiome composition and metabolism. These factors can influence the relationship between arsenic concentrations in the environment (e.g., drinking water and food) and the eventual internal arsenic body burden because the gut microbiome affects the relationship between these environments (external and internal). It is also theoretically possible that variations in microbiome composition between individuals may contribute to differences in individual susceptibility by influencing arsenic metabolite profiles. Clearly more research is needed to further characterize microbes that affect arsenic metabolism, arsenic effects on the microbiome, and links between changes in the microbiome and arsenic-associated disease outcomes.

### Modeling Aggregate Exposure

Given that arsenic is present in multiple media—food, water, soil, air, and dust—any individual is likely to have multiple routes and media of exposure. This scenario creates a substantial challenge for estimating exposure. Fate and transport, simulation, and probabilistic modeling are some approaches that can be used in conjunction with sampling measurement to estimate aggregate exposure (Dummer et al. 2015; Embry et al. 2014; Flanagan et al. 2015; Pastoor et al. 2014; U.S. EPA 2015a). These types of analyses, such as using soil sample concentrations to predict exposure and estimate health risks, are useful for risk assessment at specific sites (Gress et al. 2014). Also, some aggregate exposure modeling studies have used a multi-media, multi-pathway exposure assessment and identified house dust as an important source of exposure in mining communities (Hysong et al. 2003; O’Rourke et al. 1999).To develop a stronger foundation of data for future modeling studies, workshop participant indicated that duplicate diet studies, more sampling of food and other media, and more speciation data in all exposure media are needed to develop a stronger foundation of data for future modeling studies.

## Exposure Prevention and Mitigation Strategies

### Reducing Exposures from Water Sources

Prevention strategies to reduce exposure to arsenic from drinking water will need to address the problem from different perspectives. Strategies should consider local sources of exposure, intended use of the water supply, and the local capacity to implement the preventative strategies. There are numerous approaches to remediation of arsenic in groundwater and drinking water (Basu et al. 2014; Singh et al. 2015). Sustainable, resilient exposure prevention strategies at the local level need to account for existing community capacity and cultural norms that may affect understanding and implementation of the strategies. For example, point-of-use filters eventually filter water more slowly over time, causing people to be less likely to use them. Furthermore replacement filters are costly (Gamble M, personal communication; NIEHS 2014).

At the community level, exposure prevention requires identification of contaminated sources, notification of the problem to the community, and education to persuade people to use safer water sources. Municipal water supplies are monitored for arsenic by state or local agencies, but private wells are not. To identify local sources of exposure, particularly drinking-water wells, real-time–sensitive and affordable field detection methods that are accessible to communities are crucial. Furthermore, reliable methods that can result in greater community awareness are essential for publicizing the identity of high- and low-arsenic water sources (Balasubramanya et al. 2014; van Geen et al. 2014).

However, community awareness alone is not sufficient to affect behavior (Flanagan et al. 2015; van Geen et al. 2014). One study of 386 households in central Maine surveyed homeowners who were notified that their well water contained > 10 μg/L arsenic 3–7 years before the study. Even knowing that their water contained high arsenic concentrations, 27% of households continued to use the well water (Flanagan et al. 2015). In contrast, educating Bangladeshi elementary school children about health risks from arsenic exposure resulted in five times more families switching to cleaner wells compared with families whose children did not receive the education (Khan et al. 2015). The disparate responses point to the need for more research on factors that foster the use of prevention strategies as the best technology has no value if people do not use it.

### Reducing Dietary Exposures

The diet is an important source of arsenic exposure, and is garnering more attention as researchers seek to identify and quantify arsenic in foods (deCastro et al. 2014; Kurzius-Spencer et al. 2014; Schoof et al. 1999; Tao and Bolger 1999; Xue et al. 2010). One notable food source of arsenic is rice (Brandon et al. 2014; deCastro et al. 2014; Sauvé 2014). Given that more than half of the world’s population relies on rice for a substantial portion of their daily diet (Barker et al. 2007), it becomes essential to reduce the arsenic content of rice. One possible strategy to modify the amount of arsenic in rice plants uses plant biology and genetics. For example, studies showed that growing rice in flooded paddies made arsenic more bioavailable to rice plants than for those grown under conditions without flooding, but unflooded conditions resulted in increased cadmium uptake by the rice plant (Moreno-Jiménez et al. 2014). Other studies reported variation in arsenic uptake between different rice cultivars and genotypes (Syu et al. 2015); growing cultivars that have low arsenic uptake could potentially be a simple and cost-effective method for exposure reduction. Researchers are also using genome-wide association studies (GWAS) to identify plant genes that play a role in arsenic accumulation toward the goal of manipulating that process, either increasing absorption for soil remediation or decreasing absorption for food-source plants (Norton et al. 2014).

Levels of arsenic in the irrigation water can also be reduced using a variety of strategies. Irrigation channel dimension, water flow rate, and soil and water chemistry can all affect the effectiveness of arsenic removal from flowing irrigation water (Lineberger et al. 2013; Polizzotto et al. 2013, 2015). Several workshop participants suggested consideration of arsenic water standards set at different levels depending on intended use. For example, drinking-water standards may be more stringent than crop irrigation–water standards and yet still be protective of public health. Setting such use-specific standards requires more research to quantify exposure parameters. For irrigation-water standards, risk assessments would need to take into account plant uptake of arsenic that can vary depending on the crops and how they are grown (Chakraborty et al. 2014; Moreno-Jiménez et al. 2014). Another mitigation strategy is filtering irrigation water, as is currently used for some vineyards in Northern California (Knoll 2011).

### Reducing Soil and Dust Exposures

Soil and dust can be significant pathways of exposure, particularly near mining or smelting sites (Menka et al. 2014; Taylor et al. 2014). There are a number of different approaches to remediation of arsenic in soils and dust (Raj and Singh 2015; Singh et al. 2015; Wuana and Okieimen 2011), as well as daily-living exposure-reduction strategies such as hand and food washing (Defoe et al. 2014).

One example of a cost-effective and sustainable method to stabilize outdoor soils and dusts is phytostabilization. The goal of phytostabilization is to identify plants that could serve as permanent vegetative cover and, over time, may stabilize arsenic in the soil in a mineral form with low bioavailability. Stabilization may also reduce dispersal of contaminated dust. An ongoing study in Arizona is field testing several plants and optimizing growing conditions to maximize stabilization of arsenic-contaminated dust near a former smelting site (Valentín-Vargas et al. 2014). Recently, oxidized arsenic was co-localized with *Actinobacteria* on plant root surfaces using state-of-the-art microscopic visualization with resolution down to the 2-μm scale (Maier R, personal communication; NIEHS 2014). *Actinobacteria* are known to oxidize arsenic and to be resistant to metal toxicity (Banerjee et al. 2011), so their oxidizing capability combined with phytostabilization by the plants may provide powerful tools to reduce exposures from contaminated soils and dust; however, greater understanding of the relationships between these bacteria and the phytostabilizing plants is needed.

## Mechanisms of Response and Susceptibility to Arsenic

Mechanisms of response and biomarkers of susceptibility to arsenic are interrelated. Biomarkers can help researchers identify associated pathways and disease mechanisms. Understanding disease mechanisms can uncover new biomarkers of pathogenesis or disease precursors that may then be used to assess susceptibility in early life stages for later disease. Key data gaps lie in the links among life stage, exposure level, early effects, and later disease. Future research directions are aiming to integrate biomolecular and epidemiological data with susceptibility and health outcomes.

### Arsenic-associated Epigenetic Changes as Biomarkers and Clues to Disease Mechanisms

Emerging research on epigenetic changes following exposure to arsenic is focusing on identifying biomarkers of exposure, response, disease, and susceptibility and elucidating disease mechanisms (Ren et al. 2011). Researchers search for epigenetic changes, and hone in on loci for which the change is likely to alter gene expression. Researchers can then use a bottom-up approach to determine whether epigenetic changes for a specific gene has downstream effects on protein expression and ultimately affects the physiological response to arsenic. Identifying these pathways could in turn lead to identification of arsenic-associated health outcomes that otherwise might have been difficult to associate with arsenic exposure (Bailey et al. 2013, 2016; Bustaffa et al. 2014; Marsit 2015).

Studies are beginning to connect epigenetic changes to specific health outcomes. One study of a pregnancy cohort in Mexico screened > 400,000 CpG sites for methylation changes in 38 cord blood samples (Rojas et al. 2015). Drinking-water concentrations for this study population were 0.456–236 μg/L. They focused on 16 genes with arsenic-associated changes in methylation that also demonstrated changes in gene expression. DNA methylation levels for 7 of the 16 genes were associated with differences in gestational age and head circumference. The 16 genes are enriched for binding sites of specific transcription factors that have been shown to be altered by arsenic exposure and affect cellular signaling pathways (Rojas et al. 2015).

Researchers are also working to characterize epigenetic changes in more defined cell populations and tissues. Blood and other tissues consist of a mixture of cell types, and different cell types might have distinct epigenetic changes. For example, one study used specific differentially methylated regions (DMRs) as tags to identify specific types of blood cells in cord blood (Houseman et al. 2012). Using this technique to identify different cell subtypes, researchers examined the association between DNA methylation in cord blood and arsenic exposure via drinking water for a Bangladeshi pregnancy cohort. They found that arsenic exposure was associated with a significantly increased percentage of CD8^+^ lymphocytes and a decreased percentage of CD4^+^ lymphocytes (Kile et al. 2014). Furthermore, using the DMRs, they adjusted for the altered cell type distribution for DNA methylation analysis, and identified altered DNA methylation patterns that were associated with arsenic exposure (Cardenas et al. 2015).

More in-depth research into epigenomic, transcriptomic, and proteomic changes are needed to link changes in DNA methylation and gene expression to health outcomes. Studies need to include different lifestages, tissues, and organs as well as comparsions of response pathways at high and low doses of arsenic. Last, follow-through on linking omics data to health effects should include mechanistic studies to validate arsenic-mediated mechanisms of response.

### Identifying Susceptible Populations and Lifestages

There is ample evidence demonstrating that some individuals are more susceptible to arsenic than others. For example, exposure during early life is associated with increased risk of adverse effects that can persist into adulthood (Bailey et al. 2016; Smith et al. 2006; Steinmaus et al. 2014). One of the more striking examples is the nearly 50-fold increased standardized mortality ratio for bronchiectasis in a population of young adults in Chile who were exposed to high levels of arsenic from drinking water *in utero* and during childhood; mortality rates for this group were compared with mortality rates for the rest of the Chilean population (Smith et al. 2006). Genetic factors can also play a role in susceptibility, as demonstrated for *AS3MT* polymorphisms (Antonelli et al. 2014). As new biomarkers and factors of susceptibility are identified, as discussed above, researchers need to use that information to inform understanding of mechanisms of life stage and population susceptibility. Research is turning toward defining molecular mechanisms for these effects as well as biomarkers for susceptibility to disease in adulthood. Such diseases might be prevented or reduced through intervention in earlier life stages for susceptible individuals.

To better identify susceptible populations, susceptibility factors need to be investigated at the population level. For example, Engström et al. (2015) are analyzing *AS3MT* polymorphisms at the population level. In this study, several single nucleotide polymorphisms associated with lower urinary percent MMA occur at much higher frequency in an Argentinian Andes population living in a region where elevated arsenic levels in drinking water is common, compared with Peruvian and Colombian populations living in regions with lower arsenic level in drinking water (Schlebusch et al. 2015). These data suggest the possibility of a population adaptation to tolerate arsenic as an environmental stressor, and identify gene variants of the *AS3MT* gene associated with reduced risks. Characterization of these variants may inform strategies to minimize health effects from arsenic exposure. For example, protective variants might reveal proteins and pathways that have potential to prevent or reduce adverse arsenic-related health outcomes. Expanding on these types of studies, more large-scale genotyping of the *AS3MT* gene and other arsenic response-related genes is needed to better assess and quantitate population risks.

Clearly many factors—genetic and environmental—play a role in the response to arsenic exposure, and these factors can have various impacts in different life stages and different individuals. We are only beginning to understand how arsenic affects these processes across the life span. For example, epigenetic regulation plays an essential role in normal development whereby genes are turned on and off in sequence, and such changes are often heritable during cell division. Therefore, arsenic-associated epigenetic changes during early life may have long-term consequences. As research more clearly defines susceptibility factors, it is important to analyze those factors across populations and lifestages, and ultimately to use such information to quantitatively assess risk. The integration of molecular-level studies with *in vivo* animal and epidemiological studies is very important to delineate the importance of various susceptibility factors and identify new ones.

## Assessing and Mitigating Arsenic-associated Health Risks

Recent information about dietary exposures, newly identified health outcomes, and susceptibility factors and biomarkers provides new factors to consider in health risk assessments and risk mitigation strategies. A large number of existing data are being re-evaluated in the context of quantitative risk, but substantial data gaps remain.

### Dose Response, Susceptibility, and Cumulative Risk

Workshop participants discussed identification of key susceptibility factors that need to be evaluated in the context of risk assessment: genetics, metabolism, age, diet, and co-exposures to other agents. Research is needed on quantitation of risk with a goal of including quantifiable parameters in risk assessments. More human and rodent studies that include biomonitoring should measure arsenic species, particularly in biological samples. The percentage of MMA in urine, for example, holds promise as a quantifiable marker of risk (Engström et al. 2015; Melak et al. 2014; Pierce et al. 2013). We may then better explore possible links between percent MMA, genetic polymorphisms, and health outcomes, with the ultimate goal of linking genetic polymorphisms to quantifiable risk. More studies of humans over time are needed to better understand life stage–related risks, most especially in early life. More studies on co-exposures (e.g., metals, smoking, pesticides, asbestos, silica) could shed light on possible synergistic effects. Last, critical evaluation and new studies of arsenic effects across the full dose range, including low-dose exposure, are needed, particularly in light of new information about susceptibility biomarkers and factors.

### Nutrition and Health Risk Mitigation

Recent information about arsenic metabolic pathways and nutritional factors sheds light on the potential to use dietary changes to prevent or reduce arsenic-associated health effects. For example, researchers are focusing on the one-carbon metabolic pathway that is catalyzed by AS3MT and other enzymes, and converts arsenic into a variety of methylated species with varying toxicities (Hall and Gamble 2012). The goal is to find nutritional supplements or dietary changes that might prevent or mitigate arsenic toxicity. Researchers are now linking nutritional biochemistry studies with epidemiology to explore whether nutritional status and supplements may affect health outcomes stemming from arsenic exposure (Howe et al. 2014; Niedzwiecki et al. 2014).

Several epidemiology studies give credence to this possibility. In a Bangladeshi population, blood selenium levels were inversely associated with urinary arsenic concentrations (George et al. 2013). In another Bangladeshi population, blood levels of folate, which is used in the one-carbon metabolic pathway, were associated with arsenic methylation status in urine (Gamble et al. 2006; Howe et al. 2014). Other studies suggest that B_12_, choline, homocysteine, betaine, and creatine levels may also be associated with changes in the arsenic metabolite profile in humans (Niedzwiecki et al. 2014) and in rats (Mukherjee et al. 2006). Researchers are also studying whether nutritional supplementation can prevent or ameliorate arsenic-related health outcomes. For example, the Bangladesh Vitamin E and Selenium Trial (BEST) is a population-based, double-blind, randomized controlled trial of 7,000 adults with skin lesions that is designed to test supplementation for the prevention of nonmelanoma skin cancer (Argos et al. 2013). More research is needed to better understand the balance of nutritional influences because some nutrients at certain doses may increase arsenic toxicity, as suggested for high levels of selenium (Sun et al. 2014).

Diet may negatively affect arsenic-associated effects on fat metabolism and liver function. Previous work has shown that arsenic exposure is associated with high cholesterol, liver inflammation, and liver steatosis (Sanchez-Soria et al. 2014; Shi et al. 2014). Co-exposure to a high-fat Western diet and arsenic in mice exacerbated the effects of a high-fat diet on the liver (e.g., increased size and steatosis), resulting in degeneration that was more severe and widespread than in the controls without arsenic exposure (Sanchez-Soria et al. 2014). Mice exposed to arsenic *in utero* were affected more strongly than mice exposed later in life. Arsenic also altered the lipid metabolic product profile in a pattern that suggests disruption in the tricarboxylic acid (TCA) cycle and increased ketogenesis (Ditzel et al. 2016). Together the data suggest that the metabolism of arsenic and fatty acids are intertwined, and can impact health outcomes.

The one-carbon and TCA metabolic pathways are parts of a complex interwoven biochemical network with other biochemical pathways, and the complexity makes it difficult to predict how specific nutrients may change arsenic metabolism. Furthermore, many nutrients may affect arsenic metabolism and toxicity. A new mathematical model of arsenic metabolism has been developed to help make such predictions, and the model performed well in comparisons with epidemiological data (Lawley et al. 2014). This model can be used to predict nutritional influences on arsenic metabolism before conducting *in vivo* testing.

More data are needed to define nutritional changes and supplements that may prevent or minimize health effects. Nutritional status should be considered in epidemiological studies as a possible contributing factor to the outcomes of arsenic exposure, and collecting more data on nutrients and arsenic species in biological samples could enhance our understanding of these relationships. Because of the intricate interweaving of these biochemical pathways, it is difficult to predict how supplementation will affect the entire system. Nonetheless, nutritional supplementation holds potential as a cost-effective and practical approach to reducing health impacts of arsenic exposure.

## Conclusions

This review encompasses a number of promising research findings and future research directions related to the identification and reduction of arsenic exposures and health effects (Appendix 1). Key efforts are moving toward more detailed human aggregate exposure assessments that will require gathering information about the identification, sources, and biomonitoring of different arsenic compounds and species. The integration of technologies from multiple disciplines will be indispensable as researchers determine the complex mechanisms and develop strategies for preventing or mitigating arsenic exposure and consequent adverse health effects. A major challenge is to coalesce various data sets (e.g., omics with epidemiological and aggregate exposure data) to determine which variables are associated with the detrimental health outcomes of arsenic exposure. Finally, emerging biomarkers of arsenic exposure, effect, and susceptibility have the potential to be powerful tools for quantitative risk assessment and identification of susceptible populations and lifestages.

## Appendix 1: Summary of Important Research Needs

### Exposure Research Needs

- Assessing non-drinking-water sources of exposure, particularly diet
- Characterizing toxicokinetics and bioavailability for more arsenic species (e.g., arsenosugars, arsenolipids, arsenoproteins)
- Developing accurate and portable field testing kits for arsenic that address the challenges in sample handling and environmental arsenic detection
- Understanding relationships between biomarkers and internal exposure
- Understanding relationships between biomarkers and exposures for a greater variety of exposure media and biological tissues
- Elucidating possible health effects of co-exposure to other agents such and lead, cadmium, and fluoride
- Studying effects of arsenic on the microbiome, and effects of the microbiome on arsenic metabolism and internal exposure
- Developing approaches to estimate aggregate exposure from multiple media and multiple pathways

### Exposure Prevention and Mitigation Research Needs

- Improving remediation strategies at the local level
- Developing strategies to effect behavioral change for individuals to take action to reduce exposures
- Finding effective ways to reduce dietary exposures, particularly in rice
- Creating effective strategies to minimize arsenic exposure from soil and dust near Superfund sites

### Mechanisms of Response and Susceptibility Research Needs

- Using omics technologies to identify biomarkers linking exposure to susceptibility, disease onset, and long-term disease
- Conducting molecular epidemiology studies
- Identifying susceptible populations (e.g., life stage, genetic factors)
- Finding approaches for quantitating risks for use in risk assessments
- Exploring influences of nutrition on arsenic susceptibility and health risk reduction
